# Measuring spatial accessibility for wheelchair users: A case study in a Chinese campus

**DOI:** 10.1371/journal.pone.0335663

**Published:** 2025-12-08

**Authors:** Yizhe Huang, Chaobo Shi, Shujie Dong, Shuichao Zhang, Linghui Xu, Guangyue Nian

**Affiliations:** 1 School of Civil and Transportation Engineering, Ningbo University of Technology, Ningbo, China; 2 Zhejiang Engineering Research Center of Digital Road Construction Technology, Ningbo, China; 3 School of Transportation Science and Engineering, Civil Aviation University of China, Tianjin, China; 4 School of Smart City Management, Shanghai Construction Management Vocational College, Shanghai, China; Karolinska Institutet, SWEDEN

## Abstract

Barrier-free campus environment is essential for students and staffs with mobility impairment. However, few studies have empirically examined the spatial accessibility for wheelchair users within the campus. This study aims to investigate the travel characteristics of wheelchair users, so as to better guide the planning and design of barrier-free campus facilities. Thirty students with wheelchair experience were recruited, and their travel characteristics navigating various types of facilities were analyzed. To analyze the effect of explanatory variables on their travel speeds, a decision tree regression method was applied. Based on the empirical results, the existing pedestrian accessibility model was further extended to represent the accessibility of wheelchair users with different travel destinations. Results revealed that the type of barrier-free facility and travel mode (with or without assistance) had the most significant impact on the travel speed for wheelchair users. The average travel speed of assisted wheelchair users was 1.03 m/s, while the speed was 0.67 m/s for wheelchair users without assistance. For unassisted wheelchair users, it was challenging for them to pass through the curb ramps with slopes steeper than 1:5. The visualization results of wheelchair accessibility revealed spatial inequality within the study area, which can be further improved by upgrading existing barrier-free facilities and optimizing the allocation of different destinations.

## Introduction

In recent years, the number of people with mobility impairment is increasing. According to Centers for Disease Control and Prevention in America, 12.1% of adults have mobility disability with serious difficulty walking or climbing stairs [1]. A national survey in Canada revealed that 197,560 individuals in Canada were manual wheelchair users and 42,360 individuals were powered wheelchair users [2]. In China, a total of 85 million people have physical disabilities, with a significant proportion being people with mobility impairment [3]. For these people with mobility impairment, daily traveling is not always an easy task.

People in need of a barrier-free environment should not be neglected. Improving barrier-free environment can increase employment, education and social participation opportunities for people with disabilities [4]. In addition to people with mobility impairment, older people, pregnant women, and mothers with infant also need barrier-free facilities. However, the planning for accessible facilities remains limited in various countries. According to a review report based on eight different studies, the proportion of barrier-free wheelchair routes ranges from 14% to 76%, varying across different countries and building types [5]. A field study in Singapore indicates that, among 40 walking routes examined, only four walking routes have no barriers for wheelchair users [6]. In Japan, more than 30% of public elementary and junior high schools lack wheelchair-accessible toilets, and over 70% do not have an elevator [7]. Given the growing importance of wheelchair in urban transport, it is necessary to understand the travel characteristics of vulnerable populations (e.g., people with mobility impairment, the older people) and improve the accessible environment. Campus disability access has received considerable attention since the 1990s, particularly in the United States and Canada [8,9]. Persons with disabilities fully and equally enjoy all human rights and fundamental freedoms [10]. Although legislation and relevant building codes have been implemented, recent studies reported that a number of campuses still present structural barriers, such as the absence of elevators or ramps [11]. Students with disabilities continue to face difficulties accessing facilities [12,13]. According to a recent study in three universities campuses, accessible facilities are inadequate in providing physical access to people with mobility impairment [14]. In China, more than 50,000 disabled students were accepted in regular higher education institutions in the last five years. However, whether these students can reach their desired destinations within the campus remains unclear.

Numerous studies have examined the movement abilities of vulnerable populations, mainly focusing on emergency travel and daily travel. Table 1 provides an overview of representative studies investigating the travel behaviors of vulnerable populations across various built environments. Earlier studies primarily concentrated on understanding travel characteristics of vulnerable populations during emergencies. For example, Rubadiri et al. [15] estimated the speed of individuals with mobility impairment in obstacle-free and evacuation routes. An index called Evacuation Performance Index (EPI) was proposed to quantitatively measure and predict evacuation performance of individuals with mobility impairment. Boyce et al. [16] evaluated the movement ability of 155 individuals across different walking facilities during emergency situations, such as level ground, ramps, and stairs. Four disability groups have been categorized by the study: unassisted ambulant, unassisted wheelchair users, assisted ambulant, and assisted wheelchair users. Miyazaki et al. [17] conducted experiments to investigate travel behavior and evaluated evacuation strategies for disabled individuals. Based on the experimental results, a numerical simulation was developed to model able-bodied pedestrians overtaking wheelchair users. Kuligowski et al. [18] investigated stair evacuation speeds of vulnerable populations. By studying evacuating behaviors of 45 vulnerable residents, the overall average speeds were found to range between 0.11 and 0.29 m/s.

**Table 1:** Selected travel behavior studies of vulnerable populations.

Context	Reference	Country	Limiting condition	Sample size	Dependent variable
Emergency Travel	Rubadiri et al. (1997)	UK	Mobility	6	Speed
	Boyce et al. (1999)	UK	Mobility/ Older people	155	Speed
	Miyazaki et al. (2004)	Japan	Mobility	30	Speed
	Kuligowski et al. (2013)	US	Mobility	45	Speed
Daily Travel	Arango and Montufar (2008)	Canada	Mobility	63	Speed
	Moniruzzaman et al. (2016)	Canada	Older people	13,127	Mode choice
	Sharifi et al.(2017)	US	Visual/ Mobility	311	Speed
	Lima et al.(2019)	Brazil	Mobility	7	Speed
	Zhang et al. (2021)	China	Disabled people/Older people	Over 200,000	Travel frequency/ distance
	Willberg et al.(2023)	Finland	Older people	/	Speed

The daily travel characteristics of vulnerable populations have also been extensively investigated in the past decade or so. Arango and Montufar [19] examined crossing walking speeds of older pedestrians and found that the crossing walking speed was significantly higher than normal walking speed for older pedestrians. Moniruzzaman and Páez [20] investigated walking environment attributes from seniors’ perspectives in Montreal. The walking behavior model for seniors was estimated and a walkability audit of 403 segments was used to prove the method. Sharifi et al. [21] conducted a large-scale controlled experiment on pedestrian walking behavior involving individuals with disabilities. Individuals with disabilities’ walking speed is significantly lower than those without disabilities. Zhang et al. [22] examined the travel behaviors and activity patterns of vulnerable populations based on smart card data. The results showed that vulnerable populations exhibit distinct characteristics of travel behaviors that differ from the general population.

The low mobility of vulnerable populations poses challenges to spatial accessibility. The concept of spatial accessibility, defined as the potential of opportunities for interaction [23], is widely used in both urban planning and transportation research. Recently, an increasing number of studies have started to focus on the spatial accessibility for vulnerable populations. Lima and Machado [24] proposed an analytic hierarchy process and commented paths method to evaluate the spatial accessibility related to people with reduced mobility. Cheng et al. [25] investigated the walking accessibility to the recreation amenities for the older people and empirically estimated the adaptive distance threshold for different places. Páez et al. [26] compared the distance, time, and the metabolic energy cost for modeling walking accessibility and suggested that measuring the walking accessibility for vulnerable populations should consider their physical effort. Willberg et al. [27] measured the walking speeds of older people and proposed the walking accessibility model considering people’s walking abilities and road conditions.

Plenty of vulnerable populations, including the older people with limited mobility and individuals with mobility impairment, rely on wheelchairs to meet their mobility needs. But physical barriers hinder wheelchair users’ mobility and access to different spaces. Over the last decades, few studies have empirically examined the spatial accessibility for wheelchair users within the campus. Sahoo and Choudhury [4] explore the benefits and potential outcomes of improved wheelchair accessibility. A recently published study evaluated the spatial accessibility to healthcare services for wheelchair users. However, the travel speed of wheelchair users was assumed to be constant (1 m/s) [28], which neglected different levels of difficulty wheelchair users may encounter while navigating various facilities. This study fills the gap by investigating the travel characteristics of wheelchair users across various campus facilities, further extending spatial accessibility methods to better support the planning and design of barrier-free campus environment.

## Methods

### Field data collection

This section describes the field experiment designed for collection empirical wheelchair travel data on various barrier-free facilities. A total of 30 undergraduate students with prior experience in wheelchair use, but not as regular or long-term users, were recruited for the study. These students included 16 males and 14 females, aged 18–23. The Body Mass Index (BMI) values of these participants range from 18 to 28 kg/m^2^, with a mean value of 21.74 kg/m^2^ and a standard deviation of 2.62 kg/m^2^, indicating that they all have healthy body shapes. Recruitment was conducted via an online campus announcement targeting students with prior wheelchair using experience. Before the formal experiment, both wheelchair users and wheelchair pushers received training on maneuvering wheelchairs across different types of road facilities. It should be noted that the number of participants (30) was determined based on experience rather than statistical calculations. Similar numbers of participants have been adopted in previous related studies, such as 24 healthy participants [29], 30 healthy participants [30], and 20 healthy participants [31]. This suggests that the number of participants in our study is reasonable. Participants were recruited from March 7 to March 11, 2023. All participants provided written informed consent prior to participation. This study was exempted from review by the ethics committee.

**Fig 1:**
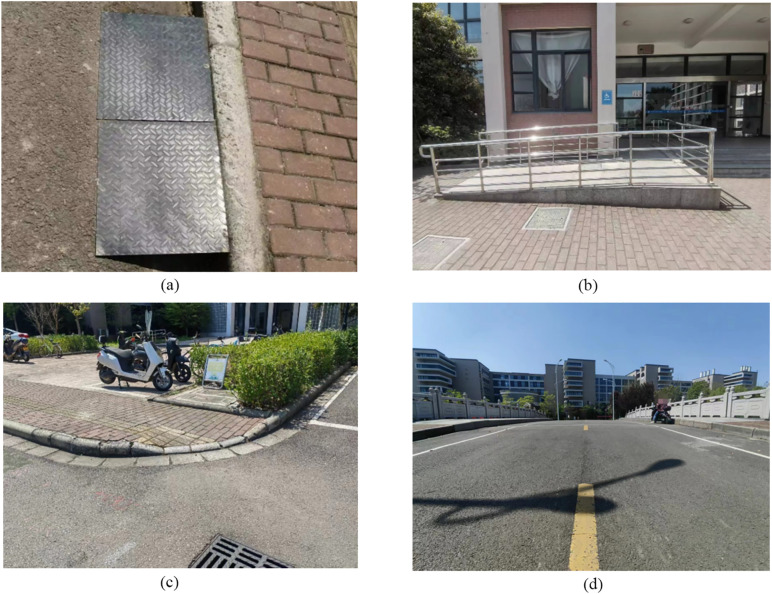
Typical barrier-free facilities. (a) Temporary curb ramp. (b) Wheelchair ramp. (c) Fixed curb ramp. (d) Accessible bridge.

The wheelchair experiments were conducted in the campus of Ningbo University of Technology. The travel characteristics of wheelchair users navigating different types of common barrier-free facilities, such as temporary curb ramp, fixed curb ramp, wheelchair ramp, outdoor pathway, and accessible bridge were measured, see Fig 1.

All participants completed a pre-survey before engaging in the experiments, including name, age, gender, weight, height, physiological and psychological conditions. For different facilities, each participant mainly participated in two types of wheelchair experiments, one with assistance from others and one without extra assistance. The experiment process was recorded using a video camera. The wheelchair travel speed and the traveling speed were calculated by measuring travel distance and travel time using the laser rangefinder and the stopwatch. The average traveling speeds of able-bodied pedestrians for each facility were also measured based on empirical observations. It should be noted that only a manual wheelchair was used for this experiment, electric wheelchairs were not within the scope of this study.

### Modeling travel speed for wheelchair users

Similar to walking speed, the travel speed for wheelchair users is influenced by various factors, such as gender, age, weather conditions, road infrastructure, and pedestrian traffic volume, which has been extensively investigated by researchers from various countries [32,33]. However, due to the limitations in sample size of wheelchair users, it is challenging to conduct large-scale field observations for the movement of wheelchair users. Based on the wheelchair experiments designed in this study, the factors influencing wheelchair travel speed are further investigated under various barrier-free facilities.

The explanatory variables in the dataset include Body Mass Index (BMI), gender (male, female), facility type (temporary curb ramp, fixed curb ramp, wheelchair ramp, outdoor pathway, accessible bridge), direction (ascend, descend, level surface), and travel mode (with assistance, without assistance). Most of the explanatory variables are categorical variables, which are not suitable for most regression models.

In this study, a decision tree regression (DTR) method is used to analyze the effect of these explanatory variables on the travel speed for wheelchair users. DTR method is a widely used predictive modeling approach in statistics and data mining, and it is well suited for categorical variables. In general, DTR selects the optimal splitting attribute through information gain, information gain ratio or Gini_index, to make sure the samples contained in the branching nodes belonging to the same category as much as possible. In this study, Gini_index is used to split the data. Assuming the proportion of samples belonging to the *n*-th class in the current sample set *D* is denoted as $p_{n}(n=1,2,...,\left|y\right|)$, then the purity of dataset *D* can be measured by Gini_index as follows:


$$Gini(D)=1-\sum_{m=1}^{\left|y\right|}p_{m}^{2}$$ (1)

A smaller value of Gini_index indicates a higher purity of the dataset. Assuming a discrete attribute a has X possible values $\left\{a^{1},a^{2},...,a^{X}\right\}$, if we use a to partition the sample set D, it will generate X branching nodes. Among them, the x-th branching node contains all the samples in D that have the attribute a with value of $a^{x}$, denoted as $D^{x}$. The Gini_index of attribute a can be defined as follows:


$$Gini\_index(D,a)=\sum_{X=1}^{X}\frac{\left|D^{X}\right|}{\left|D\right|}Gini(D^{x})$$ (2)

Thus, in the candidate explanatory variables set A, attributes that minimize the Gini_index after partitioning can be selected as the optimal splitting attributes. Based on the DTR results, the average wheelchair travel speed $V_{s}$ for various barrier-free facilities can be obtained.

### Modeling the spatial accessibility for wheelchair users

Pedestrian accessibility, defined as the ease of access by walking to desired destination [34], is broadly used in both urban planning and transportation planning fields. The term is in line with some use of “walking accessibility” or “walkability” [35,36]. By quantifying and visualizing the accessibility values of different areas, pedestrian accessibility can provide references for improving the walking environment within the region. In this study, we mainly focus on the ease of wheelchair users reaching their desired destinations. The form of spatial accessibility model for wheelchair users is extended by traditional walking accessibility models.


$$A_{im,k}=\sum_{j\in J-i}E_{j}^{k}f_{m}({T_{ij}}^{\prime }/T_{ij})f_{k}({T_{ij}}^{\prime })$$ (3)

where, $A_{im,k}$ presents the spatial accessibility for activity k at area i using travel mode m (wheelchair with/without extra assistance), $E_{j}^{k}$ is the number of activity k at area i, ${T_{ij}}^{\prime }$ is the wheelchair travel time from i to j, $T_{ij}$is the travel time from i to j at a normal walking speed. The impedance function for wheelchair users consists of two parts: the travel impedance factor $f_{k}({T_{ij}}^{\prime })$ and the wheelchair modification factor $f_{m}({T_{ij}}^{\prime }/T_{ij})$.

The travel impedance factor $f_{k}({T_{ij}}^{\prime })$ measures the proportion of people willing to reach activity k with travel time ${T_{ij}}^{\prime }$ from area i to area j*.* In line with Roper et al. [36] and Iacono et al. [37], the negative exponential function is used to represent the impedance function of spatial accessibility:


$$f_{k}({T_{ij}}^{\prime })=e^{-\beta_{k}{T_{ij}}^{\prime }}$$ (4)

where $\beta_{k}$ is the parameter of pedestrian impedance function for different activity k. The travel time for wheelchair users $T_{ij}^{\prime }$ is used as travel cost in this study. Assuming the travel route $R_{ij}$ from area i to area j has N barrier-free facilities $\left\{s^{1},s^{2},...,s^{N}\right\}$. Among them, the n -th facility s has a length of $L_{s,n}$. The average wheelchair travel speed for facility s is $V_{s,n}$, which is obtained by the previous section. Thus, the total wheelchair travel time for route $R_{ij}$ can then be calculated by adding up the cumulative travel time for various facilities as follows:


$${T_{ij}}^{\prime }=\sum_{n}L_{s,n}/V_{s,n}$$ (5)

The wheelchair modification factor $f_{m}({T_{ij}}^{\prime }/T_{ij})$ is established to modify the travel percentage of people using wheelchairs with/without extra assistance. The comparison of wheelchair travel time to normal walking travel time ${T_{ij}}^{\prime }/T_{ij}$ is used to measure the difficulty of wheelchair travel, as the indicator can affect the travel willingness of wheelchair users. The functional form of $f_{m}({T_{ij}}^{\prime }/T_{ij})$ can be empirically determined based on the experimental results.

In this study, the travel time threshold is set as $T_{ij}=$ 15 minutes, which is commonly considered as the suitable walking scale within the neighborhood [38]. For overall index of accessibility, the pedestrian accessibility for area i can then be further calculated based on the weighted sum of each type of activity k:


$$A_{im}=\sum_{k}W_{k}A_{im,k}=\sum_{k}W_{k}\sum_{j\in J-i}E_{j}^{k}f_{m}({T_{ij}}^{\prime }/T_{ij})f_{k}({T_{ij}}^{\prime })$$ (6)

where $W_{k}$ is the weighting of each type of activity *k*.

Based on Eq.(3) to Eq. (6), the detailed process of calculating the spatial accessibility for wheelchair users is shown as follows. First of all, the time spent on each facility can be calculated by dividing the length of each facility by the travel speed obtained by the DTR method. Then, the average travel time between each pair of buildings for wheelchair users ${T_{ij}}^{\prime }$ can be calculated by adding up the cumulative travel time for various facilities, see Eq. (5). The Dijkstra algorithm is applied to determine the travel route with minimum travel time. The travel impedance functions $f_{k}({T_{ij}}^{\prime })$ for different destinations and the wheelchair modification factor $f_{m}({T_{ij}}^{\prime }/T_{ij})$ can be then determined for the given travel time ${T_{ij}}^{\prime }$ and $T_{ij}$. Based on Eq.(3) and Eq.(6), by incorporating the impedance function for wheelchair users with the number of activities within different area, the wheelchair accessibility for different travel destinations and the overall wheelchair accessibility can be obtained for the research area.

## Results

### Experimental results

Each experiment was divided into two parts: one where participants used a wheelchair unassisted and another where they used a wheelchair with extra assistance. Table 2 shows the average wheelchair travel speed on various facilities. It should be noted that only barrier-free facilities are measured in this experiment. It is challenging for wheelchair users navigating through non-accessible facilities, such as stairs and arch bridges, which are prevalent on campus.

**Table 2:** Average movement speed for wheelchair users (standard deviations are in parentheses).

Facility Type	Slope	Direction	Without Assistance (m/s)	With Assistance (m/s)
Temporary Curb Ramp (TCR)	1:5	Ascending	0.00 (0.00)	0.04 (0.01)
		Descending	0.01 (0.00)	0.04 (0.01)
Fixed Curb Ramp (FCR)	1:5	Ascending	0.00 (0.00)	0.06 (0.01)
		Descending	0.01 (0.00)	0.14 (0.02)
Outdoor Pathway (OP)	0	Horizontal	0.67 (0.12)	1.03 (0.08)
Wheelchair Ramp (WR)	1:12	Ascending	0.22 (0.09)	0.82 (0.26)
		Descending	0.26 (0.03)	0.96 (0.08)
Accessible bridge (AB)	1:20	Ascending	0.41 (0.08)	1.12 (0.27)
		Descending	0.45 (0.03)	1.46 (0.04)

According to Table 2, facility type has a significant effect on travel speed (F(4, 230) = 432.62, p < 0.001). However, for curb ramps with an average slope of 1:5, there is no significant difference between fixed and temporary curb ramps (p = 0.9998). For unassisted wheelchair users, it is difficult to pass through curb ramps with this slope, and the wheelchair speed is close to 0 m/s. At the same time, travel mode (with assistance, without assistance) also has a significant effect on travel speed (F(1, 230) = 430.17, p < 0.001). Outdoor pathway is the most common walking facility without any slope. The average travel speed for unassisted wheelchair users is 0.67 m/s, and the speed is 1.03 m/s for wheelchair with assistance. This result is in line with another observation study of wheelchair travel characteristics [39]. Compared to the slope of curb ramp (1:5), the average slope of the wheelchair ramp is gentler (1:12). Most wheelchair users are able to ascend or descend the wheelchair ramp without extra assistance. The slope of accessible bridge in this study is 1:20. The average travel speed of unassisted wheelchair users is 0.41–0.45 m/s when passing through the accessible bridge, while the speed is 1.12–1.46 m/s for wheelchair with assistance. It should be noted that facility type and travel mode have a significant interaction effect (F(4, 230) = 65.92, p < 0.001). The effect of extra assistance depends on the facility type. For example, with extra assistance of others, the speed of pushing a wheelchair through an accessible bridge is faster than through an outdoor pathway. The main reason is that pushing the wheelchair to pass through an accessible bridge requires substantial energy expenditure, and pushing slowly may lead to increased physical exertion. In order to pass through the accessible bridge as quickly as possible, some participants ran a short distance before getting on the bridge.

### Results of DTR method

The experimental data were recorded across five different types of facilities (temporary curb ramp, fixed curb ramp, wheelchair ramp, outdoor pathway, and accessible bridge), two travel modes (with assistance and without assistance), and three directions of wheelchair travel (ascending/descending for ramps and the bridge, and horizontal for the outdoor pathway). Each of the 30 participants completed 18 wheelchair trials. Since curb ramps with an average slope of 1:5, whether fixed or temporary, could not be passed without assistance, the unassisted ascending and descending trials on both curb ramps were excluded. As a result, each participant contributed 14 valid data samples and a total of 420 data points were obtained, containing information on name, age, gender, weight, height, facility type, travel mode, direction, and travel speed.

During the decision tree regression process, 80% of the data (336 samples) was used for model training, and the remaining 20% (84 samples) was used to test the prediction accuracy of the model [40].

The DTR method has a good model fit using the experimental data. The R^2^ of the model is 0.932 and the mean square error (MSE) is 0.014. The goodness-of-fit measures were also employed to evaluate the prediction performance of DTR using the testing data. In this study, mean error (ME), mean percent error (MPE), and root mean square error (RMSE) were used to quantify the overall error of the model results [41]. For testing data, the goodness-of-fit measures ME = 0.02, MPE = 21.3%, and RMSE = 0.12, indicating that the DTR method has a reasonable representation of the wheelchair travel speed data.

The variable importance values are calculated based on the DTR method, see Table 3. The results show that the type of barrier-free facility (temporary curb ramp, fixed curb ramp, wheelchair ramp, outdoor pathway, accessible bridge) and travel mode (with assistance, without assistance) have the most significant impact on the travel speed for wheelchair users. The direction of wheelchair travel (ascend, descend, level surface) also has an impact on the movement speed, although the impact is relatively weaker. Individual characteristics (e.g., BMI and gender) are eliminated by the DTR method due to their insignificant impacts on the wheelchair speed (Relative Importance <1). The reason for this may be that the participants are healthy undergraduate students with similar ages and physical conditions, resulting in non-significant effects of these variables. Additionally, the experiment involved only short-distance movement, which made differences in endurance and strength less likely to influence wheelchair speed.

**Table 3:** Variable importance.

Explanatory Variable	Relative Importance
Facility type	53.61
Travel mode	40.97
Direction	5.41

**Fig 2:**
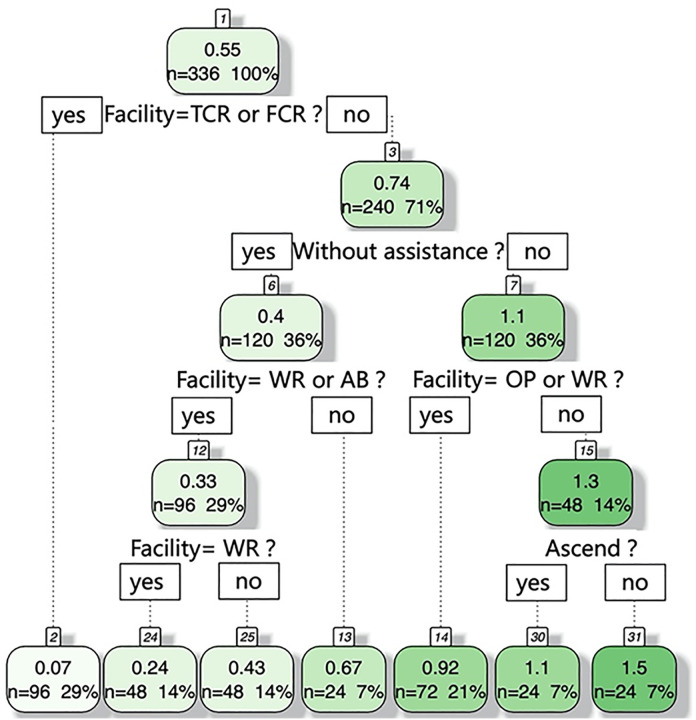
Results of the DTR method.

Fig 2 shows the decision tree created by the experimental data. The first node divides the data based on whether the barrier-free facility is temporary curb ramp (TCR) or fixed curb ramp (FCR). Whether assisted or not, it is challenging for most wheelchair users to across the curb ramp in this experiment. In the second level node, the data is divided based on whether the wheelchair users are assisted by others. The third and fourth level node of this decision tree are divided based on the facility types (OP, WR, AB) and direction (ascending, descending). Most of the results in Fig 2 are consistent with the average movement speed for wheelchair users shown in Table 2. Without assistance, the average travel speeds for wheelchair ramp (0.24 m/s) and accessible bridge (0.43 m/s) are slower than the outdoor pathway (0.67m/s). With extra assistance, the average travel speed for wheelchair to across the wheelchair ramp and outdoor pathway is 0.92 m/s. The speed of pushing wheelchair across an accessible bridge is faster than outdoor pathway (Ascend: 1.1m/s; Descend: 1.5m/s).

### Results of spatial accessibility for wheelchair users

#### Estimation results of impedance functions for wheelchair users.

A set of travel impedance functions $f_{k}({T_{ij}}^{\prime })$ for different types of destinations are extracted from the pedestrian impedance functions measured by Iacono et al. [37], which are shown in Table 4. In this study, travel time ${T_{ij}}^{\prime }$ is the measure of travel cost for wheelchair users, units are in minute. The dependent variable y measures the faction of trips for a given wheelchair travel time ${T_{ij}}^{\prime }$.

**Table 4:** Summary of travel impedance functions for different destinations [37].

Impedance Function	School	Work	Restaurant	Recreation
	$y=.\text{5}\text{2}\text{4}e^{-.\text{1}\text{0}\text{6}T_{ij}^{\prime }}$	$y=.\text{5}\text{1}\text{1}e^{-.\text{1}\text{0}\text{6}T_{ij}^{\prime }}$	$y=.\text{3}\text{7}\text{3}e^{-.\text{0}\text{9}\text{3}T_{ij}^{\prime }}$	$y=.\text{5}\text{5}\text{6}e^{-.\text{1}\text{0}\text{0}T_{ij}^{\prime }}$

The travel impedance functions in Table 4 are estimated for pedestrians without mobility impairment. In this study, for wheelchair users, the travel impedance is modified by the passage difficulty of each wheelchair route. According to the experimental results, for a given travel time ${T_{ij}}^{\prime }$ for wheelchair users, the extra time taken for wheelchair travel compared to normal walking travel can substantially decrease the number of wheelchair trips.

**Fig 3:**
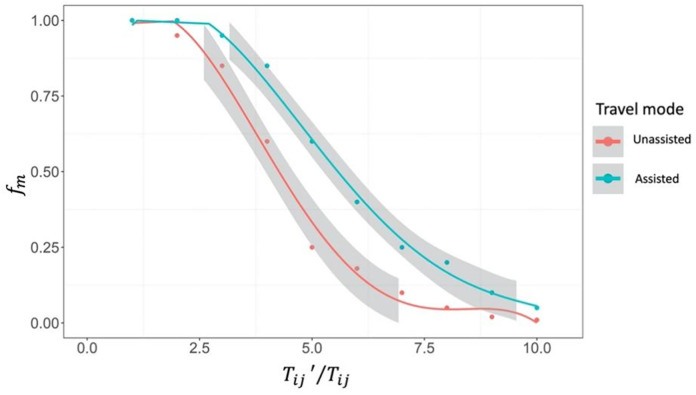
Experimental results of wheelchair modification factor ${𝐟}_{m}$.


In this study, the wheelchair modification factor $f_{m}({T_{ij}}^{\prime }/T_{ij})$ is used to modify the faction of trips. Based on the experimental results, the polynomial functions are applied to fit the relationships between the extra time for wheelchair users ${T_{ij}}^{\prime }/T_{ij}$ and trip modification factor $f_{m}$. As presented in Fig 3, the polynomial function shows a good fit to the data (adjusted R-squared > 0.95). The fitting results of trip modification factor $f_{m}$ are shown as follows:

$$\{\begin{array}{c} {}*20c-0.0013*{\left(\frac{{T_{ij}}^{\prime }}{T_{ij}}\right)}^{4}+0.0307*{\left(\frac{{T_{ij}}^{\prime }}{T_{ij}}\right)}^{3}-0.2403*{\left(\frac{{T_{ij}}^{\prime }}{T_{ij}}\right)}^{2}+0.5220*\frac{{T_{ij}}^{\prime }}{T_{ij}}+0.6800\;\;\;\;\;\;\;\;\;\;unassisted\;person\\ -0.0005*{\left(\frac{{T_{ij}}^{\prime }}{T_{ij}}\right)}^{4}+0.0153*{\left(\frac{{T_{ij}}^{\prime }}{T_{ij}}\right)}^{3}-0.1470*{\left(\frac{{T_{ij}}^{\prime }}{T_{ij}}\right)}^{2}+0.3955*\frac{{T_{ij}}^{\prime }}{T_{ij}}+0.7208\;\;\;\;\;\;\;\;\;\;assisted\;person \end{array}$$ (7)

#### Visualization results of spatial accessibility for wheelchair users.

**Fig 4:**
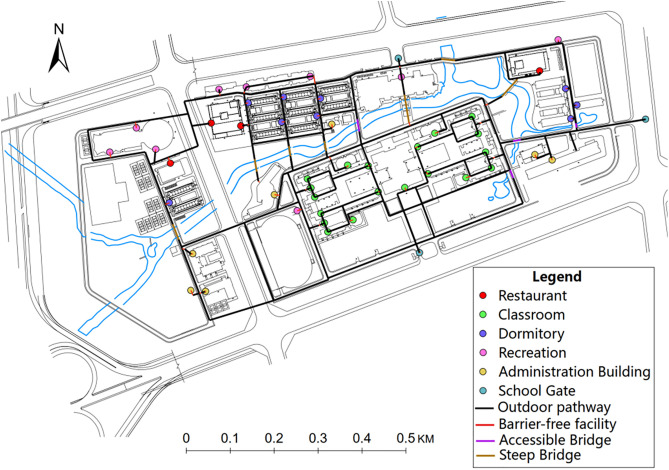
The map of study area.

To illustrate the procedures used to estimate the spatial accessibility for wheelchair users, a visualization results of spatial accessibility for wheelchair users is presented in a typical university campus in China. The campus covers an area of 6.74 km^2^ and most buildings can be reached within a 15-minute walk from the central point of the campus. Fig 4 shows the general layout of study area. Different colors of spots represent various facility types within the campus, including restaurant, classroom, dormitory, recreation, and administration building. The barrier-free facilities (temporary curb ramp, fixed curb ramp, wheelchair ramp) are mainly located at the entrance of each building. The outdoor pathways within the campus, shown as solid black lines on the map, are accessible for wheelchair users. The campus is intersected by a river, with a total of 11 bridges. Among them, four bridges are accessible bridges, while seven bridges are arch bridge with steep slope (exceed 1:10).

**Fig 5:**
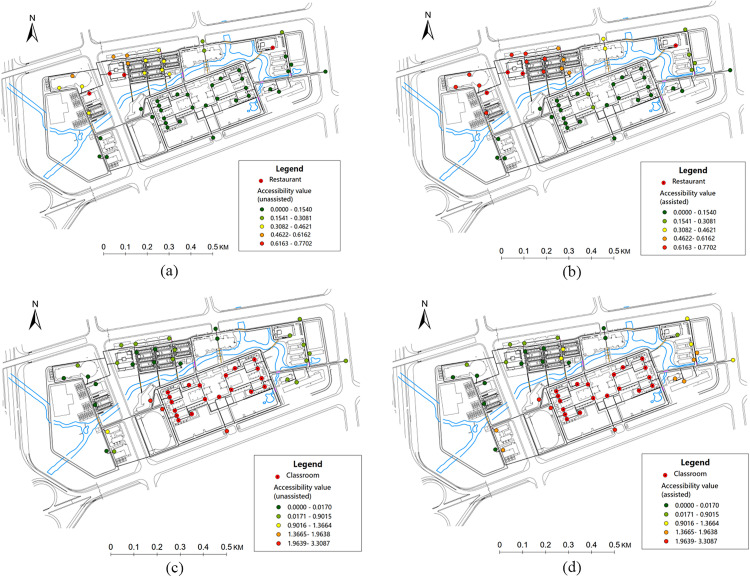
Wheelchair accessibility for different travel destinations. (a) Wheelchair accessibility to restaurant without assistance. (b) Wheelchair accessibility to restaurant with extra assistance. (c) Wheelchair accessibility to classroom without assistance. (d) Wheelchair accessibility to classroom with extra assistance.

For students and staffs in the campus, attending class and having meals are two primary activities. The wheelchair accessibility to the restaurants and classrooms within the research area are calculated for the research area, see Fig 5. As shown in Fig 5, the northwestern region of the campus exhibits higher accessibility to restaurants for students and staffs using wheelchair. Areas near clusters of classrooms (the central region of the campus) exhibits higher level of wheelchair accessibility to classrooms. The accessibility for students and staffs using wheelchairs can be substantially improved with assistance from others. Based on the accessibility results, for wheelchair users, the school can place them in the dormitories located in the central-northern region of the campus, which provides better accessibility for dining and attending classes.

**Fig 6:**
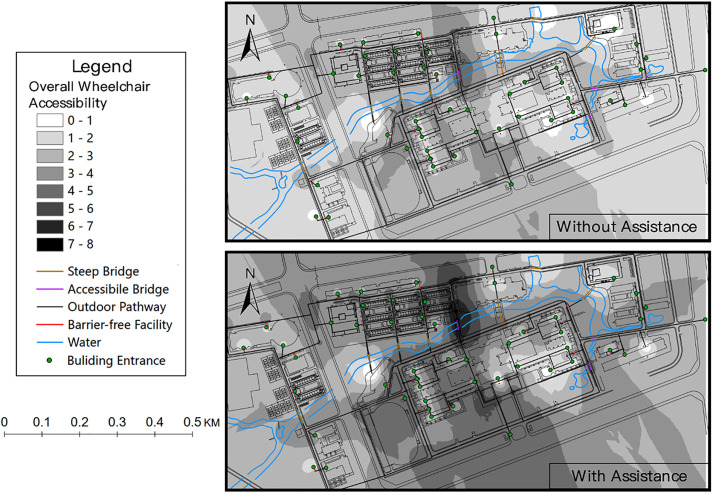
The overall wheelchair accessibility within the campus.

Fig 6 presents the overall wheelchair accessibility within the campus. In this case, the values of accessibility to various destinations are measured for each pedestrian network junction and building entrance. The weighting of each type of destination is assumed to be equal in this case. Consistent with the results in Fig 5, the central-northern region of the campus exhibits high levels of wheelchair accessibility due to its location advantages, a diverse range of facility types, and well-maintained barrier-free facilities. However, there are still facilities that are inaccessible, such as steep bridges (brown lines in the figure), significantly hinders students and staffs using wheelchairs.

These two examples illustrate the role that barrier-free facilities and distribution of various types of activities play in determining wheelchair accessibility. The maps of wheelchair accessibility provide both the objectives and a quantitative evaluation method for improving wheelchair accessibility in the target area. Based on the results, the accessibility for wheelchair users can be improved by upgrading existing barrier-free facilities and adding various types of facilities within the study area, or by placing wheelchair users in favorable locations within the campus. For a comprehensive campus master planning, the following recommendations can be provided: 1) The barrier-free facilities should be planned at the network level, as the discontinuous accessible routes can substantially decrease the accessibility of wheelchair users. 2) It is recommended to replace most arch bridges and curb ramps with low-slope barrier-free facilities, such as wheelchair ramp and accessible bridge. 3) Different functional areas on campus should not be planned to be too dispersed. Mixed-use developments on land can provide a convenient environment for wheelchair users. 4) The dormitory for wheelchair users can be arranged in areas close to the classrooms and restaurants.

## Discussion

In this study, the travel characteristics of wheelchair users were empirically investigated through a series of wheelchair experiments. According to the results of decision tree regression, the type of barrier-free facility and travel mode (with assistance, without assistance) had the most significant impact on the travel speed for wheelchair users. These findings are consistent with numerous relevant studies, indicating that steeper slopes substantially increase physical exertion, thereby reducing travel speed for wheelchair users [29,30,42]. Compared with unassisted wheelchair users, those who used a wheelchair with extra assistance could travel faster and cover greater distances, which is in line with Levy et al. [31]. It is found that the direction of wheelchair travel (ascending, descending, level surface) can slightly influence movement speed of wheelchair users, which is consistent with Kim et al. [43]. However, in certain facility types, such as the accessible bridge and the wheelchair ramp, descending speeds were not significantly higher than those on outdoor pathways, which was mainly due to participants’ safety concerns.

Individual characteristics (e.g., BMI and gender) were eliminated in the DTR analysis due to their insignificant impacts on the wheelchair speed. This finding aligns with Kim et al. [43], who reported that average travel speed was not significantly affected by the wheelchair user’s body weight or the assistant’s strength, mainly because people tended to prioritize safety when operating the wheelchair. Likewise, another study found no significant difference in wheelchair travel speed between males and females, although males generally have greater strength than females [44]. Based on the results of wheelchair experiments, the spatial accessibility model is further proposed for wheelchair users with/without extra assistance from others. The wheelchair accessibility for different travel destinations and the overall wheelchair accessibility are empirically estimated within the study area. The maps of wheelchair accessibility illustrate the role that barrier-free facilities and distribution of various types of activities play in determining wheelchair accessibility. To the best of our knowledge, few studies have examined accessibility for wheelchair users. The existing pedestrian accessibility literature provides a methodological foundation and supports our findings [45,46].

## Conclusions

This study provides a quantitative evaluation method for improving the spatial accessibility of wheelchair users on campus. The results show that the type of barrier-free facility and the travel mode significantly influence the travel speed for wheelchair users. The direction of wheelchair travel can slightly influence travel speed. Individual characteristics such as BMI and gender are found to have negligible effects. Based on the visualization results of wheelchair accessibility, spatial accessibility for wheelchair users in different campus areas can be substantially enhanced by upgrading existing barrier-free facilities and adding various types of facilities.

Though this study provides a new perspective on the spatial accessibility method for wheelchair users, several limitations must be noted. First, the sample size of wheelchair experiment is limited and the participants in the experiment were all students. As a result, the research findings may not be representative of a more diverse population. Second, this study only measured wheelchair speeds on some representative barrier-free facilities. The impact of design parameters of facilities, such as different slope and paving material, on the wheelchair speeds can be further investigated in the future. Third, only manual wheelchairs were used in this study, and no comparison was made with computerized wheelchair users. Given that computerized wheelchairs may exhibit different travel speeds on barrier-free facilities, this lack of comparison may limit the generalizability of the findings to all wheelchair user groups. Finally, this study only conducted an empirical analysis of wheelchair accessibility in a university campus. Since the proposed wheelchair accessibility method is also applicable to other urban areas, a city-scale analysis of wheelchair accessibility can be conducted in the future.
